# Cell-Free DNA 5-Hydroxymethylcytosine Signatures for Lung Cancer Prognosis

**DOI:** 10.3390/cells13040298

**Published:** 2024-02-06

**Authors:** Jianming Shao, Randall J. Olsen, Saro Kasparian, Chuan He, Eric H. Bernicker, Zejuan Li

**Affiliations:** 1 Department of Pathology and Genomic Medicine, Houston Methodist Hospital, Houston, TX 77030, USA; 2 Houston Methodist Research Institute, Houston, TX 77030, USA; 3 Weill Cornell Medical College, New York, NY 10065, USA; 4 Cancer Center, Houston Methodist Hospital, Houston, TX 77030, USA; 5 Department of Medical Oncology, City of Hope Comprehensive Cancer Center, Duarte, CA 91010, USA; 6 Department of Chemistry, Department of Biochemistry and Molecular Biology, Institute for Biophysical Dynamics, The University of Chicago, Chicago, IL 60637, USA; 7 Howard Hughes Medical Institute, The University of Chicago, Chicago, IL 60637, USA

**Keywords:** cell-free DNA, 5-hydroxymethylcytosine, prognosis, lung cancer

## Abstract

Accurate prognostic markers are essential for guiding effective lung cancer treatment strategies. The level of 5-hydroxymethylcytosine (5hmC) in tissue is independently associated with overall survival (OS) in lung cancer patients. We explored the prognostic value of cell-free DNA (cfDNA) 5hmC through genome-wide analysis of 5hmC in plasma samples from 97 lung cancer patients. In both training and validation sets, we discovered a cfDNA 5hmC signature significantly associated with OS in lung cancer patients. We built a 5hmC prognostic model and calculated the weighted predictive scores (wp-score) for each sample. Low wp-scores were significantly associated with longer OS compared to high wp-scores in the training [median 22.9 versus 8.2 months; *p* = 1.30 × 10^−10^; hazard ratio (HR) 0.04; 95% confidence interval (CI), 0.00–0.16] and validation (median 18.8 versus 5.2 months; *p* = 0.00059; HR 0.22; 95% CI: 0.09–0.57) sets. The 5hmC signature independently predicted prognosis and outperformed age, sex, smoking, and TNM stage for predicting lung cancer outcomes. Our findings reveal critical genes and signaling pathways with aberrant 5hmC levels, enhancing our understanding of lung cancer pathophysiology. The study underscores the potential of cfDNA 5hmC as a superior prognostic tool for guiding more personalized therapeutic strategies for lung cancer patients.

## 1. Introduction

Lung cancer is one of the most common cancers and the leading cause of cancer-related deaths in the United States [1]. Prognostic assessment plays a crucial role in guiding clinical management and informing treatment decisions for lung cancer patients. A prognostic biomarker is measured before treatment and provides information on long-term outcomes irrespective of therapeutic interventions. Currently, TNM (tumor, node, metastasis) classification is a conventional biomarker used for prognosticating lung cancer but lacks accuracy due to imaging modalities and interpretation [2,3,4]. Other factors, including patient age, sex, and performance status, also play roles in non-small cell lung cancer (NSCLC) outcomes [3,5,6]. Gene mutations have been widely used as predictive biomarkers for targeted therapy [7]. Some genes, such as *EGFR*, *KRAS*, and *TP53*, are reported to be associated with poor prognosis in NSCLC patients [8,9,10]. However, the prognostic value of these gene mutations is debatable [8,9,10]. Notably, several studies have highlighted the prognostic significance of gene-specific promoter DNA methylation in lung cancer [11,12,13,14,15]. However, existing markers often lack precision or practical clinical applicability [4,12]. Thus, there is a critical need for the development of novel and accurate prognostic markers to improve lung cancer patient outcomes.

An emerging epigenetic marker in the field of cancer research is 5-hydroxymethylcytosine (5hmC) [16,17,18,19,20,21]. 5hmC is an intermediate product of DNA demethylation, generated through the oxidation of 5-methylcytosine (5mC) by ten-eleven-translocation proteins [22]. It is predominantly distributed in genomic regions such as enhancers, promoters, and gene bodies, and its levels are positively correlated with gene expression and inversely correlated with alterations in 5mC [16,17,18,19,22]. Dynamic changes in 5hmC have been associated with cancer initiation, progression, metastasis, and prognosis in various cancers, including lung cancer [16,17,23,24,25,26,27,28,29,30,31,32]. Notably, reduced global 5hmC levels in tumor tissue have been linked to poor prognosis in lung cancer patients [33].

A recently developed and highly selective method, nano-hmC-Seal, enables the precise mapping of genome-wide 5hmC distributions in plasma cell-free DNA (cfDNA) [34]. CfDNA are degraded DNA fragments released into body fluids from both normal and tumor cells in cancer patients. Several studies, including our own, have shown that cfDNA 5hmC is a highly sensitive marker for the early detection of lung cancer and other cancer types [16,17,18,19,20,21,35,36,37,38,39,40,41,42,43,44]. Our group and others also demonstrated that a cfDNA 5hmC signature is significantly associated with patient prognosis in acute myeloid leukemia and pancreatic cancer [19,44]. However, the prognostic potential of 5hmC in cfDNA for lung cancer remains unexplored, and the role of 5hmC in lung cancer prognosis is not well understood.

To investigate the prognostic value of cfDNA 5hmC in lung cancer patients, we used nano-hmC-Seal combined with next-generation sequencing (nano-hmC-Seal-Seq) to profile the genome-wide distribution of 5hmC in 97 plasma cfDNA samples from stage I to IV lung cancer patients. Our study revealed a 5hmC signature significantly associated with the survival of lung cancer patients. This cfDNA 5hmC signature outperformed traditional prognostic factors in predicting patient outcomes and represents the first use of cfDNA 5hmC signatures for lung cancer prognosis. We also identified novel genes and signaling pathways with aberrant 5hmC levels that impact lung cancer prognosis.

## 2. Materials and Methods

### 2.1. Patients and Sample Collection

We collected blood samples from 97 adult patients with lung cancer at Houston Methodist Hospital from 2013 to 2022 (Table 1). These patients ranged in age from 50 to 92 years (median 70 years). The lung cancer subtypes include NSCLC (*n* = 95) and small-cell lung cancer (*n* = 2). The subtypes of NSCLC included adenocarcinoma (*n* = 65), squamous cell carcinoma (*n* = 23), and other forms (*n* = 7). TNM stages included Stage I (*n* = 22), Stage II (*n* = 7), Stage III (*n* = 11), and Stage IV (*n* = 57). Thirty-one patients received lobectomy or wedge resection, with 13 providing blood samples post-surgery and the rest at diagnosis or pre-surgery. Ten patients received no treatment after sampling, while others underwent chemotherapy (carboplatin, pemetrexed, paclitaxel, and/or etoposide), targeted therapy with tyrosine kinase inhibitors (erlotinib, osimertinib, alectinib, and cabozantinib), or immune checkpoint inhibitor treatment.

### 2.2. Study Design

We conducted genome-wide sequencing of 5hmC in plasma cfDNA samples from all 97 participants. To establish a 5hmC prognostic signature, we randomly divided the cfDNA samples into a training set (*n* = 58) and a validation set (*n* = 39) in a 6:4 ratio. We assessed the correlation between 5hmC distribution and overall survival (OS) in the training set and developed a 5hmC signature significantly associated with OS. Based on this signature, we constructed a 5hmC prognostic model and calculated a weighted prognostic score (wp-score) for each sample to represent the 5hmC levels of the signature genes. We established a specific cutoff wp-score to distinguish between patients with longer and shorter OS. We validated the model in the validation set. Additionally, we explored genes and canonical signaling pathways with aberrant 5hmC levels significantly associated with lung cancer prognosis.

OS was defined as the time from registration to death or last follow-up. Progression-free survival (PFS) was defined as the time from registration to tumor progression, therapy change, or death from any cause, with censoring for patients lost to follow-up. TNM staging was based on the 8th edition of the TNM Classification for lung cancer [45]. Disease progression was assessed using the Response Evaluation Criteria in Solid Tumor guidelines, version 1.1 [46]. This study was approved by the Houston Methodist Hospital Institutional Review Board.

### 2.3. Plasma Preparation and DNA Extraction

Peripheral blood samples were collected in Vacutainer EDTA tubes (BD, Franklin Lakes, NJ, USA). Plasma was isolated from 3 mL of blood through centrifugation at 4 °C, 1350× *g* for 10 min, and subsequently stored at −80 °C. cfDNA was extracted from approximately 1 mL of plasma samples using the QIAamp Circulating Nucleic Acid Kit (QIAGEN, Germantown, MD, USA). The quantity of cfDNA was measured using the Qubit 4.0 fluorometer and the Qubit dsDNA HS Assay Kit (Thermo Fisher Scientific, Waltham, MA, USA). The quality of the cfDNA was assessed using the Agilent High Sensitivity DNA Kit and the Bioanalyzer 2100 (Agilent Technologies, Santa Clara, CA, USA).

### 2.4. hmC Profiling and Sequencing Data Processing

The 5hmC library was constructed following previously established protocols [18]. Briefly, the cfDNA underwent end repair and adaptor ligation. Next, 5hmC-containing DNA fragments were enriched using T4 bacteriophage β-glucosyltransferase, DBCO-PEG4-biotin (Sigma, St. Louis, MO, USA), and streptavidin beads (Thermo Fisher Scientific, Massachusetts). The enriched library was sequenced using 2 × 100 paired-end sequencing reads on the NovaSeq 6000 instrument (Illumina, San Diego, CA, USA). Sequencing data were processed as previously described with minor modifications [18]. We evaluated the quality of the raw reads using FastQC (https://www.bioinformatics.babraham.ac.uk/projects/fastqc/; accessed on 1 May 2020) and trimmed adaptors and low-quality reads using Trimmomatic, version 0.32. High-quality raw reads were mapped to the human reference genome (GRCh38) using bowtie2, version 2.4.5, with the end-to-end mode. Reads with a mapping quality score ≥ 20, insert size < 600 bp, ≤1 ambiguous base, and <3 mismatches were retained. High-quality mapped reads were counted into gene bodies without strand information using the RefSeq database with featureCounts software, version 2.0.0. Preliminary quality control steps filtered genes and calculated counts per million reads (CPM) for library size normalization. Genes with CPM < 3 in over half of the samples were removed from downstream analysis.

### 2.5. Development of 5hmC Prognostic Signatures

To create the 5hmC prognostic signatures, we followed a methodology similar to that described in a previous study [19]. We correlated 5hmC levels with OS in the training set using a univariate Cox proportional-hazards regression model. To reduce dimensionality and remove uninformative markers, we selected genes significantly associated with OS (*p* < 0.05) for downstream analysis. Subsequently, we conducted feature selection by applying elastic net regularization with an α range from 0.55 to 0.95 in increments of 0.1 to a multivariate Cox proportional-hazards model using the glmnet package, version 4.0. Hyperparameters were optimized using 10-fold cross-validation with the cv.glmnet function using the Harell C index from the glmnet R package. This process was iterated 100 times to identify robust gene signatures. Genes that appeared in at least 95% of iterations were considered signature genes for the final 5hmC model development. Seventeen genes were analyzed using a multivariate Cox proportional hazards model. To quantify the wp-scores for the best prognostic model, we employed the following formula: wp-score $=\sum_{k=1}^{n}\left(\beta_{k}\times gene_{k}\right)$, where *β_k_* is the coefficient for the *k*th marker gene from the final multivariable Cox proportional hazards model, and *gene_k_* is the normalized 5hmC level of the *k*th marker gene, as previously described [19]. The specific cutoff for the wp-scores was determined using the surv_cutpoint function from the survminer R package (https://github.com/kassambara/survminer; accessed on 26 July 2022) in the training set.

### 2.6. Statistical Analyses

We utilized the Kaplan–Meier estimator to estimate PFS and OS over time in our patient cohort. Survival differences between groups were assessed using the log-rank test from the R package ‘survival’ (https://cran.r-project.org/web/packages/survival/index.html; accessed on 1 August 2022). Hazard ratios (HRs) were calculated using the Cox proportional-hazards regression model, allowing us to evaluate the impact of different variables on survival outcomes between different groups. We assessed the association between the wp-score and OS while accounting for various factors using a multivariate Cox proportional-hazards regression analysis. The performance of our 5hmC prognostic model was evaluated using the ‘timeROC’ package in R, version 0.4 [47]. This analysis allowed us to calculate the area under the curve (AUC) of the receiver operating characteristic (ROC) curve for the best-fit model. A larger AUC value indicates superior model performance. We generated Forest plots using the ‘forestplot’ package (https://cran.r-project.org/web/packages/forestplot/index.html; accessed on 1 September 2023) to visualize the association between individual factors and survival outcomes. All statistical tests and data visualization were performed using R language version 4.1.1. We performed gene enrichment analyses using Ingenuity Pathway Analysis to identify significant pathways and biological processes associated with genes that exhibited aberrant 5hmC levels. Dot plots were generated using the ggplot2 package in R (https://cran.r-project.org/web/packages/ggplot2/index.html; accessed on 1 July 2021). A *p*-value of less than 0.05 was considered statistically significant for all analyses.

## 3. Results

### 3.1. A 5hmC Signature Is Significantly Associated with Overall Survival in Lung Cancer Patients

We performed genome-wide profiling of 5hmC in 97 plasma cfDNA samples obtained from lung cancer patients. Using a machine learning approach, we randomly split cfDNA samples into a training and validation set. In the training set, we correlated genome-wide 5hmC distribution with OS and identified 252 genes with aberrant 5hmC levels significantly associated with OS (*p* < 0.05). We then performed feature selection and discovered 17 genes that comprised our prognostic signature (Supplementary Table S1). These genes were deemed the most relevant and robust indicators of survival outcomes in lung cancer patients based on their consistent association with OS during the feature selection process. We developed a weighted prognostic model based on the prognostic signature and calculated a wp-score. Based on their wp-scores, a cutoff score of 310.6 was determined to differentiate between different prognostic categories, such as high-risk and low-risk patient groups.

**Figure 1:**
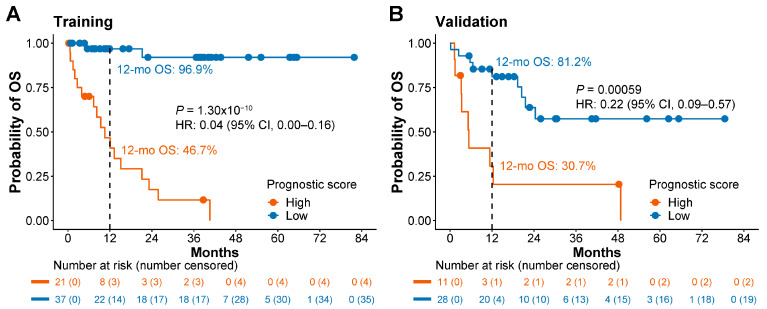
The 5hmC prognostic signature is associated with overall survival in lung cancer patients. (**A**,**B**) Kaplan–Meier analysis of overall survival (OS) based on weighted prognostic scores in the training set (**A**) and the validation set (**B**). A cutoff score of 310.6 was used for different prognostic categories. HR, hazard ratio. CI, confidence interval.

In the training set, patients with low wp-scores exhibited a significantly longer OS (median, 22.9 months) compared to patients with high wp-scores [median, 8.2 months; *p* = 1.30 × 10^−10^; HR 0.04; 95% confidence interval (CI), 0.00–0.16]. Furthermore, the 12-month OS rate was 96.9% for patients with low prognostic scores, indicating a favorable prognosis, compared to a 46.7% 12-month OS rate for patients with high prognostic scores (Figure 1A).

The validation set confirmed the robustness of the 5hmC prognostic model. Patients with low wp-scores continued to exhibit a significantly longer median OS of 18.8 months, compared to 5.2 months for patients with high wp-scores (*p* = 0.00059; HR 0.22; 95% CI: 0.09–0.57; Figure 1B). The 12-month OS rate for patients with low prognostic scores was 81.2%, reflecting a favorable prognosis, but 30.7% for patients with high prognostic scores (Figure 1B).

### 3.2. The 5hmC Signature Is Significantly Associated with PFS in Lung Cancer Patients

**Figure 2:**
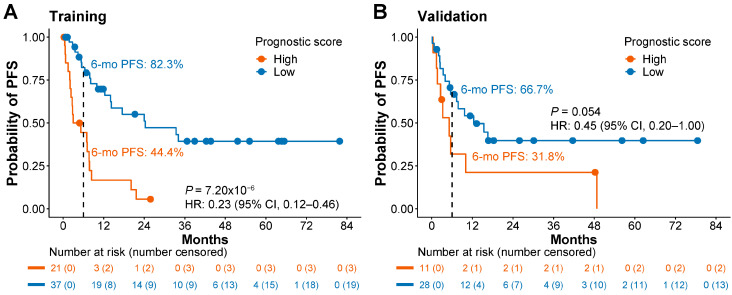
The 5hmC prognostic signature is associated with progression-free survival in lung cancer patients. (**A**,**B**) Kaplan–Meier analysis of progression-free survival (PFS) based on prognostic scores in the training set (**A**) and the validation set (**B**). A cutoff score of 310.6 was used for different prognostic categories. HR, hazard ratio. CI, confidence interval.

To extend the applicability of the 5hmC prognostic signature beyond OS to PFS, we correlated the wp-scores with PFS for lung cancer patients. In the training set, patients with low wp-scores exhibited a median PFS of 12.3 months, and patients with high wp-scores had a significantly shorter median PFS of 3.0 months (*p* = 7.2 × 10^−6^; HR 0.23; 95% CI, 0.12–0.46; Figure 2A). Additionally, the six-month PFS rates were notably different between the two groups: 82.3% in patients with low prognostic scores versus 44.4% in patients with high prognostic scores (Figure 2A).

In the validation set, patients with low wp-scores exhibited a substantially longer median PFS of 8.8 months, compared to 3.3 months for patients with high wp-scores (Figure 2B). Although the *p*-value in this case was marginally significant (*p* = 0.054; HR 0.45; 95% CI, 0.20–1.0; Figure 2B), the data supported an association between low wp-scores and improved PFS. The six-month PFS rates were notably different between the two groups: 66.7% in patients with low prognostic scores versus 31.8% in patients with high prognostic scores (Figure 2B).

### 3.3. The 5hmC Signature Is an Independent Predictor for Prognosis in Lung Cancer

**Figure 3:**
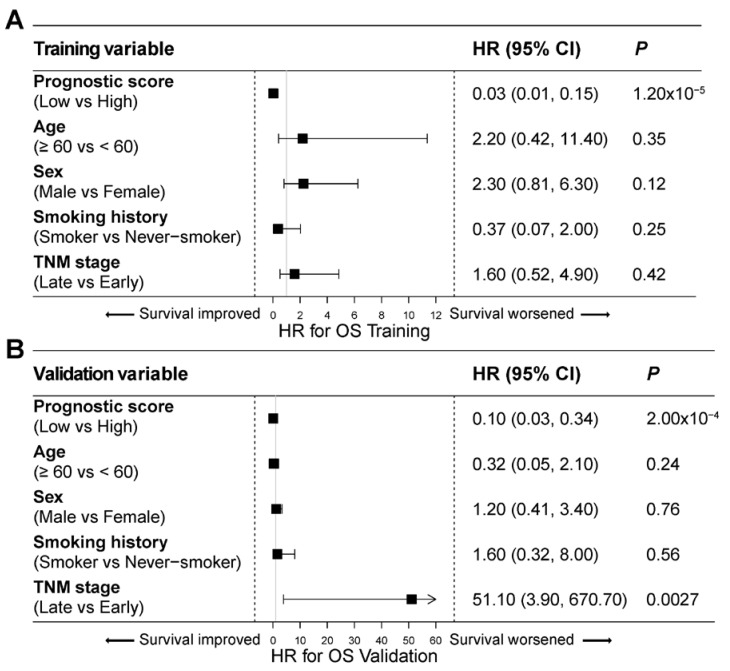
Multivariate Cox regression analysis in lung cancer patients. (**A**,**B**) Overall survival multivariate Cox regression analysis, illustrated as a forest plot, considering various clinical parameters in the training set (**A**) and validation set (**B**) of lung cancer patients. HR, hazard ratio. CI, confidence interval.

We performed multivariate Cox regression analysis to assess the independent predictive power of the cfDNA 5hmC signature in the context of other well-established clinical factors, including age, sex, smoking history, and TNM stage. In both the training (*p* = 1.2 × 10^−5^; Figure 3A) and validation (*p* = 2.0 × 10^−4^; Figure 3B) sets, the 5hmC prognostic score consistently demonstrated significant predictive power for patient prognosis, independent of TNM stage, age, sex, and smoking history. These results validate the 5hmC signature as an independent predictor for prognosis.

### 3.4. The 5hmC Signature Outperforms Other Prognostic Predictors in Lung Cancer

**Figure 4:**
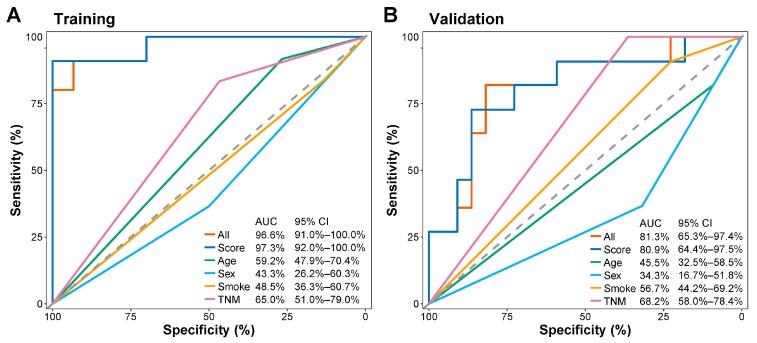
Prognostic value of multiple variables in lung cancer patients. (**A**,**B**) Time-dependent receiver operating characteristic (ROC) and corresponding area under the curves (AUCs) for 12-month overall survival predicted by all combined factors, prognostic score, age, sex, smoking history, and TNM stage in the training (**A**) and validation (**B**) sets.

Comparative analysis using time-dependent ROC revealed that the 5hmC prognostic score was more accurate in predicting prognosis than age, sex, smoking, or TNM stage in both the training and validation sets (Figure 4A,B). In the training set, the AUC for the 5hmC signature was 97.3% (95% CI, 92.0–100.0%; Figure 4A) and 80.9% (95% CI, 64.4–97.5%; Figure 4B) in the validation set. Integrating the 5hmC prognostic score with other clinical factors in the validation set slightly improved predictive ability, resulting in an AUC of 81.3% (95% CI, 65.3–97.4%; Figure 4B).

### 3.5. The 5hmC Signature Is Significantly Associated with Clinical Outcomes in Different Subtypes of Lung Cancer

As adenocarcinoma and squamous cell carcinoma are the most common subtypes of NSCLC, we evaluated whether the cfDNA 5hmC signature was associated with these subtypes. No significant differences were observed in wp-scores between the two subtypes in either the training (*p* = 0.84) or validation (*p* = 0.28) sets (Supplementary Figure S1). We then analyzed the prognostic value of the 5hmC signature for each subtype individually. Patients with adenocarcinoma with low wp-scores exhibited a significantly longer median OS of 16.7 months, compared to 9.0 months for patients with high wp-scores (*p* = 3.3 × 10^−7^; HR 0.15; 95% CI, 0.07–0.35; Supplementary Figure S2A). Similarly, patients with squamous cell carcinoma with low wp-scores also showed a significantly longer median OS of 40.1 months, compared to 5.7 months for patients with high wp-scores (*p* = 5.9 × 10^−6^; HR 0.03; 95% CI, 0.00–0.27; Supplementary Figure S2B). Moreover, low wp-scores were significantly associated with a longer PFS in both the adenocarcinoma (median 10.4 versus 5.0 months, *p* = 4.6 × 10^−4^; HR 0.35; 95% CI, 0.19–0.65; Supplementary Figure S2C) and squamous cell carcinoma (median 16.6 versus 2.7 months, *p* = 6.5 × 10^−5^; HR 0.09; 95% CI, 0.02–0.37; Supplementary Figure S2D).

### 3.6. Genes and Pathways Associated with Lung Cancer Prognosis

**Figure 5:**
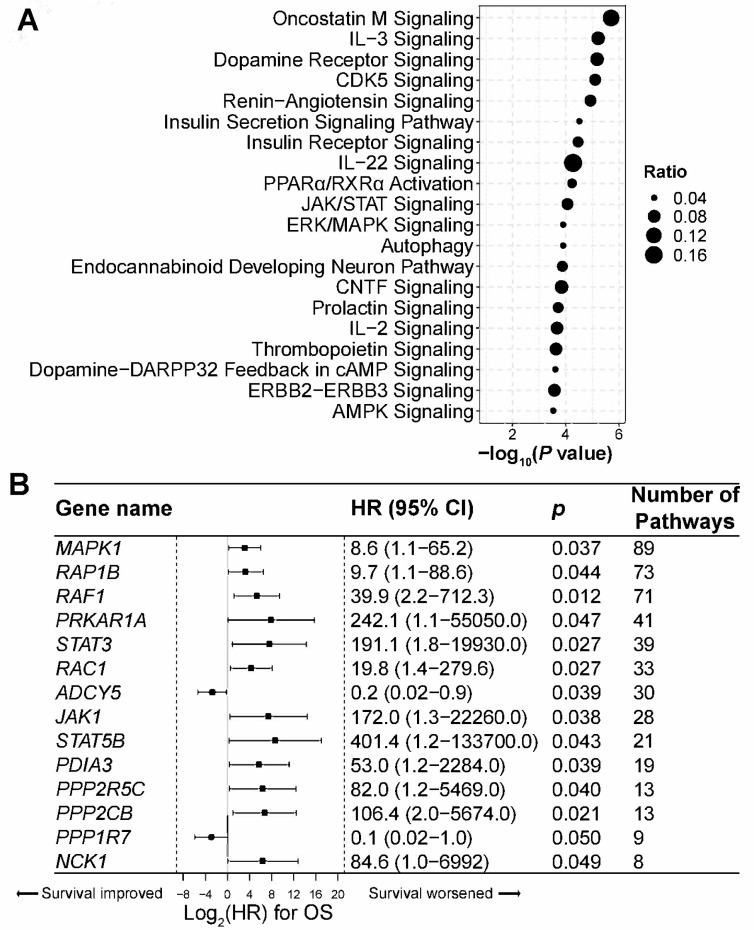
Genes and pathways associated with the prognosis of lung cancer. (**A**) Canonical signaling pathways with genes significantly associated with overall survival (OS) in lung cancer. Pathway analysis was performed using Ingenuity Pathway Analysis. The ratio indicates the number of OS-related genes in each pathway divided by the total number of genes that make up that pathway. (**B**) Genes appearing in more than 10 canonical pathways are displayed. Hazard ratios for OS in genes significantly enriched in canonical pathways presented by the forest plot.

The 5hmC analysis provided crucial insights into the genes and pathways influencing lung cancer prognosis. Our comprehensive gene enrichment and pathway analyses spanned 252 genes in which 5hmC levels were significantly associated with OS in lung cancer patients. Notably, these genes were significantly enriched in 180 canonical signaling pathways (Supplementary Table S1). Among these, pathways related to cell proliferation, such as Oncostatin M, JAK/STAT, and ERK/MAPK, were prominent, as were cytokine signaling pathways, such as IL-3, IL-22, and IL-2 (Figure 5A; Supplementary Table S1). Key genes, including *MAPK1*, *RAP1B*, and *RAF1,* featured prominently in these pathways, underscoring their functional relevance in lung cancer (Figure 5B; Supplementary Table S1). Fourteen genes were present in over ten canonical pathways. Among them, twelve genes, including *MAPK1*, *RAP1B*, and *RAF1*, were associated with shorter OS, while two genes (*ADCY5* and *PPP1R7*) were associated with longer OS (*p* < 0.05; Figure 5B).

## 4. Discussion

This study demonstrates the cfDNA 5hmC prognostic signature as an independent predictor of OS in lung cancer patients. The multifaceted and complex nature of lung cancer, arising from both inherent biology and environmental exposures, makes variables such as age, sex, smoking status, and TNM stage important determinants in patient prognosis [2,3,4,5,6]. However, our findings reveal that the cfDNA 5hmC signature outperforms these factors for lung cancer prognosis with an AUC of 80.9%, which surpasses the respective values of 45.5%, 34.3%, 56.7%, and 68.2% associated with the aforementioned predictors (Figure 3B). Further, integrating the 5hmC signature with these clinical variables may enhance its prognostic sensitivity, highlighting its potential as a standalone or adjunctive tool in prognostic assessment and guiding treatment strategies.

The TNM staging system is widely used for clinical prognosis assessments in lung cancer patients but relies heavily on tumor and lymph node imaging. This reliance introduces variability due to differing imaging techniques and the interpretive skills of readers [4], who may often underestimate tumor sizes, leading to potential false negatives or false positives [4]. Additionally, the accuracy of TNM assessments can be impacted by various factors [2], thereby limiting the accuracy of prognosis in lung cancer. TNM staging also typically requires biopsies to confirm suspected metastases and surgical resection for pathologic cancer staging [2,4]. In contrast, the less invasive cfDNA 5hmC markers provide a more objective alternative to the TNM staging system.

cfDNA 5hmC markers offer significant advantages over other research markers in predicting lung cancer prognosis. Novel prognostic biomarkers focused on protein expression, gene mutations, or DNA methylation often lack consistency across studies [3]. 5hmC provides a more faithful representation of disease status due to its preferential distribution in gene bodies and its superior reflection of gene expression compared to 5mC [48]. The cfDNA 5hmC approach also eliminates the need for bisulfite treatment, preserving high-quality sequencing data for accurate predictions [34]. Considering that 5hmC is present at only 1–10% of the abundance of 5mC [17], and with the nano-hmC-Seal method enabling further enrichment of 5hmC-containing DNA fragments, the 5hmC approach requires fewer sequencing reads. This results in reduced sequencing costs compared to genome-wide DNA methylation analysis. Further, while DNA methylation studies typically require tumor tissue [11,12,13,14,15], cfDNA 5hmC markers offer a convenient and non-invasive approach that accurately reflects the dynamic tumor landscape.

Compared to the use of gene mutations for lung cancer prognosis, the 5hmC approach may be applicable to a broader patient population. Driver mutations in genes, including *EGFR*, *ALK*, *ROS1*, *RET*, *BRAF*, and *KRAS*, are critical predictors for targeted therapy in NSCLC patients [7]. However, the prognostic implications of these mutations are often limited, and there is an ongoing debate about the reliability of *EGFR*, *ALK*, *ROS1*, and *KRAS* for accurately predicting prognosis in NSCLC patients [8,9]. *TP53*, a tumor suppressor gene, is mutated in 40–50% of lung cancer patients but portrays an unclear relationship: though many studies observed a worse prognosis in lung cancer patients with a *TP53* mutation, others indicated no significant impact on survival [8,10]. Epigenetic alterations, prevalent in all patients, significantly contribute to lung cancer development and progression [11], whereas gene mutations occur in only about half of lung cancer cases [49]. Notably, the abundance of 5hmC modification loci in the cancer genome far exceeds that of gene mutations [17,50], potentially providing higher sensitivity for detection and a more comprehensive reflection of disease status in a larger patient cohort. Further studies are needed to precisely compare these approaches and explore potential synergies by combining the 5hmC approach with other markers for enhanced prognostic efficacy.

Circulating tumor DNA (ctDNA) levels reflect tumor burden and are associated with prognosis in lung cancer patients. Typically, this approach involves analyzing somatic mutations in tumor samples and subsequently monitoring the dynamic profile of these mutations in plasma cfDNA after treatment [51]. The presence of ctDNA could predict disease relapse in these patients [51]. However, unlike the cfDNA 5hmC marker, the ctDNA approach is not a suitable source of prognostic information at the time of diagnosis. Moreover, its applicability is limited in approximately half of lung cancer patients, as not all patients exhibit somatic mutations in their tumors that can be tracked for monitoring. A comparative analysis between the cfDNA 5hmC marker and the ctDNA approach for predicting lung cancer prognosis has not yet been conducted.

As a prognostic biomarker that provides information on disease recurrence, PFS, and OS in cancer patients irrespective of the treatment they receive [52], the 5hmC signature could be used for risk stratification of lung cancer patients in clinical settings once it is validated in prospective studies. This stratification could lead to tailored treatment regimens based on identified risk groups. For example, patients in a low-risk group, such as those with surgically respectable tumors, may not require additional adjuvant therapy after tumor removal. Conversely, high-risk patients may benefit from additional adjuvant treatments to mitigate the risk of tumor recurrence. Adjustment of treatment strategies based on prognostic biomarkers has demonstrated improved survival rates in other cancers, such as colon cancer [52].

cfDNA 5hmC markers have demonstrated remarkable versatility across various applications, exhibiting high sensitivity and specificity in pan-cancer detection [16,17,18,19,20,21,35,36,37,38,39,40,41,42,43] and the ability to differentiate cancer types and tissue origins [18,48]. This utility extends to predicting prognosis in various cancers, such as acute myeloid leukemia [19], pancreatic cancer [44], and lung cancer, as demonstrated herein. Consequently, a single blood sample assay can be used to analyze distinct 5hmC signatures/markers for cancer detection, origin confirmation, and prognosis prediction. This streamlined approach significantly alleviates test burden and enhances convenience for patients.

The role of 5hmC in lung cancer prognosis remains incompletely understood. Here, we demonstrate that genes significantly associated with prognosis are enriched in pivotal pathways relevant to lung cancer, including JAK/STAT and ERK/MAPK signaling pathways. Notably, cytokine signaling pathways, including IL-3, IL-22, and IL-2, were also prominently featured. We identified genes with abnormal 5hmC levels linked to lung cancer prognosis. For example, elevated 5hmC levels in *MAPK*s like *MAPK1* and *RAF1* were significantly associated with shorter OS, consistent with their recognized oncogenic roles in cancer [53]. This finding aligns with current research on pharmacological inhibition of MAPK pathway genes (i.e., *BRAF*, *KRAS*, and *MEK1/2*) for lung cancer treatment, highlighting the clinical relevance of altered 5hmC levels in these genes [53]. Additionally, our finding of increased 5hmC in *RAP1B*, a GTP-binding protein associated with poor prognosis in multiple cancers, including lung cancer [54], indicates potential new directions for therapy, including the use of hypomethylating agents. These insights underscore the potential importance of these pathways in lung cancer prognosis. Specifically, understanding aberrant 5hmC changes in *RAP1B* and other genes enriches our understanding of the molecular mechanisms driving lung cancer tumorigenesis, paving the way for novel targeted therapeutic approaches.

Our study acknowledges certain limitations. Firstly, while we validated the 5hmC prognostic signature in the validation set, broader validation through multicenter prospective studies with more diverse patient populations is essential before considering clinical applications. Secondly, as the 5hmC prognostic signature was developed from a patient cohort at a single institution, its validation in a broader population is warranted to ensure its applicability across varied clinical settings. Thirdly, combining the 5hmC prognostic signature with other prognostic markers not included in this study may potentially improve the accuracy of lung cancer prognosis.

## 5. Conclusions

In summary, our study presents compelling evidence that plasma cfDNA 5hmC signatures serve as a potent, highly sensitive, and minimally invasive tool in lung cancer management, offering a non-invasive and effective approach to prognostication. The critical genes and signaling pathways with aberrant 5hmC levels identified herein enhance our understanding of lung cancer pathophysiology. The clinical significance of the 5hmC signature is highlighted by its effectiveness as a robust prognostic marker, accurately distinguishing between lung cancer patients with high and low survival probabilities. In clinical settings, the 5hmC signature could be used to guide clinical management and treatment decisions, enabling more personalized and effective treatment strategies and ultimately contributing to improved patient outcomes. Plasma cfDNA 5hmC markers offer a safe, simple, and non-invasive approach to lung cancer prognosis and treatment planning. 

## Figures and Tables

**Table 1:** Patient demographics and baseline characteristics.

Characteristics	All Patients N = 97
Age, median (range), y	70 (50–92)
Female, No. (%)	54 (55.7)
ECOG PS, No. (%)	
0	11 (11.3)
1	50 (51.5)
2	2 (2.1)
3	2 (2.1)
4	3 (3.1)
Unavailable	29 (29.9)
Smoking status, No. (%)	
Current	22 (22.7)
Former	57 (58.8)
Never	18 (18.6)
Histology, No. (%)	
NSCLC	95 (98)
Adenocarcinoma	65 (67.0)
Squamous	23 (23.7)
Other subtypes	7 (7.2)
Small cell	2 (2)
Disease stage, No. (%)	
I	22 (22.7)
II	7 (7.2)
III	11 (11.3)
IV	57 (58.8)
PD-L1 expression, No. (%)	
<1%	36 (37.1)
≥1%	29 (29.9)
Unavailable	32 (33.0)
PFS median, mo (range)	7.7 (0.2–81.8)
OS median, mo (range)	13.3 (0.2–81.8)

ECOG PS, Eastern Cooperative Oncology Group performance status score; NSCLC, non-small cell lung cancer; PFS, progression-free survival; OS, overall survival; mo, months.

## Data Availability

The raw 5hmC sequencing data are available in the National Center for Biotechnology Information Gene Expression Omnibus database, GSE202988 and GSE237087.

## Supplementary Materials

The following supporting information can be downloaded at: https://www.mdpi.com/article/10.3390/cells13040298/s1, Figure S1. Association of the 5hmC signature with subtypes of lung cancer. Figure S2. The 5hmC prognostic signature is associated with survival in lung cancer subtypes. Table S1. List of genes associated with prognosis in lung cancer.
