# Supplementary material for: Cell-Free DNA 5-Hydroxymethylcytosine Signatures for Lung Cancer Prognosis
Source: Cells. 2024 Feb 6;13(4):298. doi: 10.3390/cells13040298 (PMC10886903; doi:10.3390/cells13040298)
Supplement: Supplementary file 1 [file cells-13-00298-s001.zip › Supplementary_Cells_R_F.pdf]

Cell-Free DNA 5-Hydroxymethylcytosine Signatures for Lung Cancer Prognosis

Jianming Shao, Randall J. Olsen, Saro Kasparian, Chuan He, Eric H. Bernicker and Zejuan Li

Contents

Table S1. List of genes associated with prognosis in lung cancer. ………..…………. Attachment

Figure S1. Association of the 5hmC signature with subtypes of lung cancer. …………………….2

Figure S2. The 5hmC prognostic signature is associated with survival in lung cancer subtypes. 3

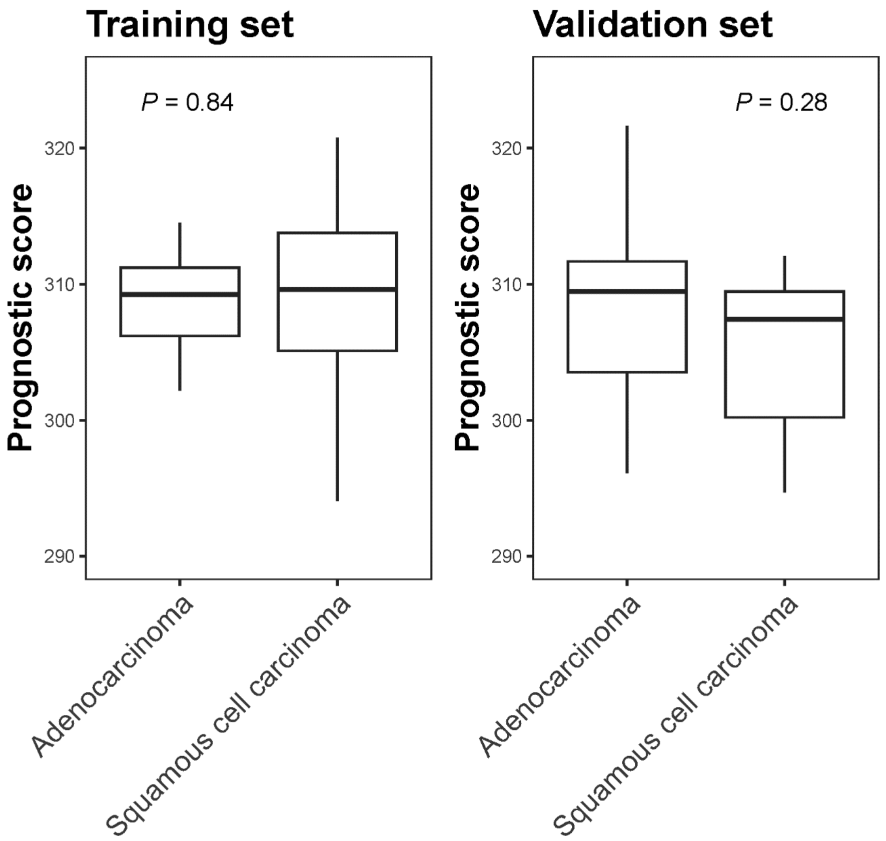

Figure S1. Association of the 5hmC signature with subtypes of lung cancer. Distribution of prognostic scores (wp-scores) in patients with adenocarcinoma or squamous cell carcinoma of lung cancer in the training and validation sets. Thirty-six patients with adenocarcinoma and 16 patients with squamous cell carcinoma were included in the training set. Twenty-nine patients with adenocarcinoma and five patients with squamous cell carcinoma were included in the validation set. Center line represents median, bounds of box represent 25th and 75th percentiles, and whiskers are Tukey whiskers.

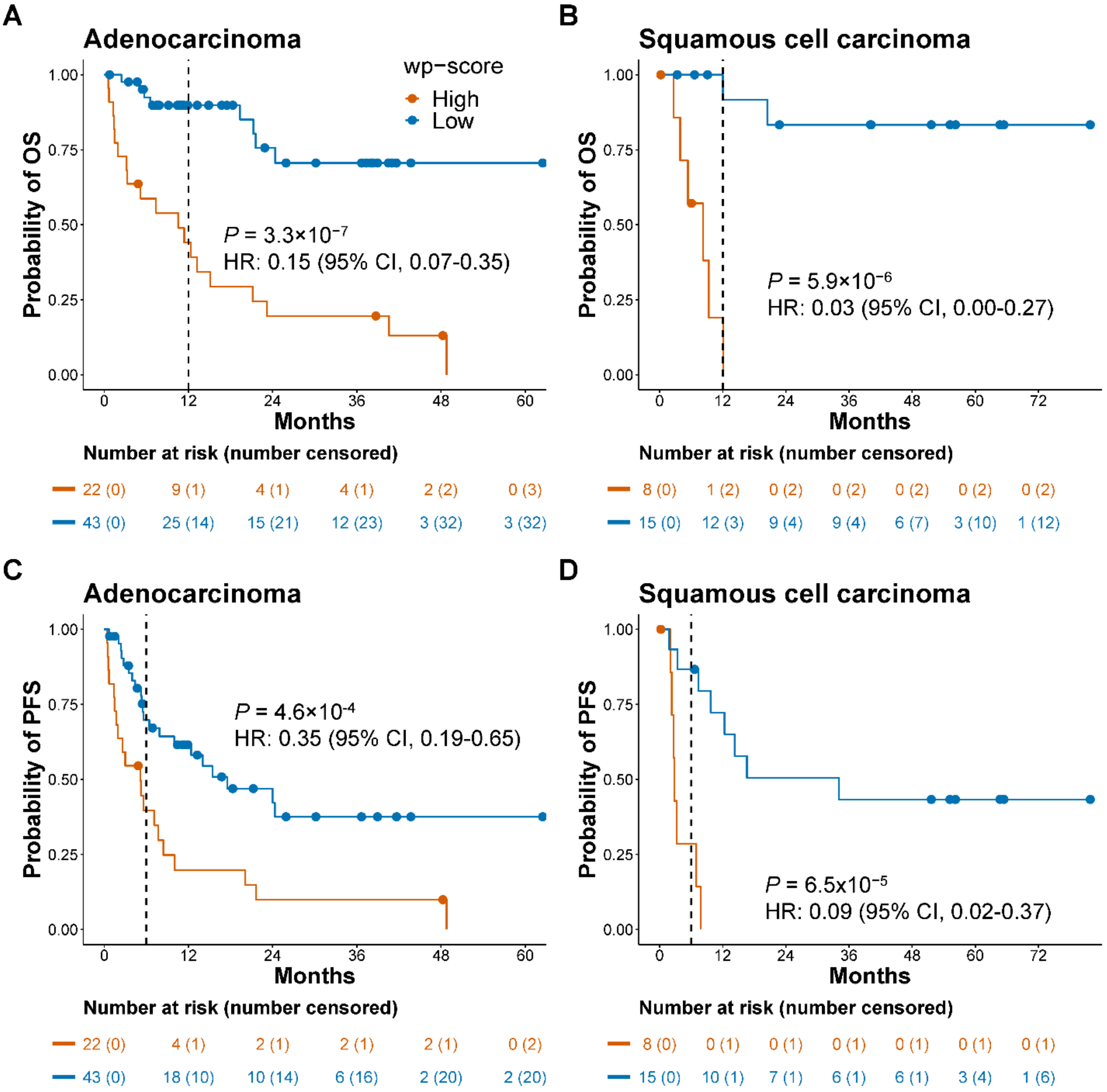

Figure S2. The 5hmC prognostic signature is associated with survival in lung cancer subtypes. (A) Kaplan-Meier analysis of overall survival (OS) based on weighted prognostic scores (wp-score) in adenocarcinoma patients. (B) Kaplan-Meier analysis of OS based on wp-scores in squamous cell carcinoma patients. (C) Kaplan-Meier analysis of progression-free survival (PFS) based on wp-scores in adenocarcinoma patients. (D) Kaplan-Meier analysis of PFS based on wp-scores in squamous cell carcinoma patients. A cutoff score of 310.6 was used for different prognostic categories. HR, hazard ratio. CI, confidence interval.
